# Concerns about covert HIV testing are associated with delayed presentation of suspected malaria in Ethiopian children: a cross-sectional study

**DOI:** 10.1186/1475-2875-13-301

**Published:** 2014-08-06

**Authors:** Yusuf Haji, Wakgari Deressa, Gail Davey, Andrew W Fogarty

**Affiliations:** 1 School of Public Health, College of Health Sciences and Medicine, Wolaita Sodo University, Wolaita Sodo, Ethiopia; 2 Department of Preventative Medicine, School of Public Health, College of Health Sciences, Addis Ababa University, Addis Ababa 6850, Ethiopia; 3 Brighton and Sussex Medical School, Brighton, UK; 4 Division of Epidemiology and Public Health, University of Nottingham, Nottingham, UK

**Keywords:** Malaria, HIV, Diagnosis, Delay, Stigma, Ethiopia

## Abstract

**Background:**

Early diagnosis is important in preventing mortality from malaria. The hypothesis that guardians’ fear of covert human immunodeficiency virus (HIV) testing delays presentation of children with suspected malaria was tested.

**Methods:**

The study design is a cross-sectional survey. The study population consisted of guardians of children with suspected malaria who presented to health centres in Oromia Region, Ethiopia. Data were collected on attitudes to HIV testing and the duration of children’s symptoms using interview administered questionnaires.

**Results:**

Some 830 individuals provided data representing a response rate of 99% of eligible participants. Of these, 423 (51%) guardians perceived that HIV testing was routinely done on blood donated for malaria diagnosis, and 353 (43%) were aware of community members who delayed seeking medical advice because of these concerns. Children whose guardians suspected that blood was covertly tested for HIV had longer median delay to presentation for evaluation at health centres compared to those children whose guardians did not hold this belief (three days compared to two days, p < 0.001). Children whose guardians were concerned about covert HIV testing were at a higher odds of a prolonged delay before being seen at a health centre (odds ratio 1.73, 95% confidence intervals: 1.10 to 270 for a delay of ≥3 days compared to those seen in ≤2 days).

**Conclusion:**

Children whose guardians believed that covert testing for HIV was routine clinical practice presented later for investigation of suspected malaria. This may account for up to 14% of the delay in presentation and represents a reversible risk factor for suboptimal management of malaria.

## Background

Malaria constitutes an important treatable cause of global morbidity and mortality, with 3.4 billion people estimated to be at risk of infection in 2012, resulting in approximately 200 million cases of malarial infection and 600,000 deaths, predominantly in young children [1]. Malaria is one of the most important public health problems in Ethiopia, representing the commonest communicable disease and accounting for 30% of the overall disability adjusted life years lost [2] as well as imposing a high economic cost [3]. As malaria can be diagnosed using laboratory assays and effective treatments exist for malaria [4], the national malaria control programme states the importance of improving treatment-seeking behaviour for fever [2]. However, introducing diagnostic algorithms that involve new rapid diagnostic blood tests for malaria can be problematic [5], although delayed presentation is a substantial problem in treating malaria [6-8], and the identification of any barriers that impede presentation at health care facilities is a priority area of research as these may be amenable to intervention.

The prevalence of human immunodeficiency virus (HIV) infection in Ethiopia in 2011 was estimated to be 4.2% in urban areas and 0.6% in rural areas [9]. There is a stigma associated with HIV infection [10-12], and 25% of pregnant women in Gambella region refused antenatal HIV testing in 2008 [13]. Hence, despite the availability of treatment, individuals may be reluctant to undergo testing of their HIV status, as they do not wish to be exposed to the knowledge and social consequences of a potential positive diagnosis.

Despite the requirement for informed consent before requesting an HIV test on any blood sample, there may be a perception that having a blood test for malaria is synonymous with being tested for HIV infection, as previously reported in qualitative studies elsewhere [14-16]. This is important, as it is a perception that is open to modification by appropriate educational programmes, hence improving the early delivery of appropriate anti-malarial treatments to patients. Hypothesizing that individuals’ concerns about covert HIV testing is impeding presentation of symptomatic children for investigation and treatment for malaria, a cross-sectional observational study design in Ethiopia to test this hypothesis in children with suspected malaria visiting health centres.

## Methods

### Study population

The study design is a cross-sectional survey. This study was conducted in East Shewa Zone of Oromia Regional State in Ethiopia. The total population of the Zone in 2010/11, as projected based on the 2007 census, was 1,519,103 (51% males and 49% females). The Zone is located in the Great Rift Valley of East Africa and malaria is a major health problem in the area. Five health centres, namely Modjo, Meki, Batu, Bulbula, and Shashemenne, were included in the study and data were collected in October and November 2012. All children under the age of 16 years who presented with symptoms consistent with malaria and gave finger-prick blood for microscopic examination at the health centres and their guardians constituted the study population. Data were collected opportunistically, when the guardian was available for interview. The study protocol was approved by the University of Nottingham Ethics Committee and the Addis Ababa University School of Public Health Research and Ethics Committee. All participants gave informed consent and received standard treatment for their presenting condition.

### Data collection

A pre-tested structured questionnaire initially developed in English and then back translated into local language (*Afan Oromo*) was used for data collection (see Additional files 1 and 2). The main sections of the questionnaire comprised of sociodemographic characteristics, concerns about HIV testing and treatment-seeking behaviour for the sick child. One data collector and one supervisor from each health centre were trained on the data collection instrument, interview techniques and recruitment of the study participants. Subsequent diagnosis of malaria was done using blood film examination under microscopy.

### Statistical analysis and power calculation

All completed data collection forms were examined for completeness and consistency during data management, storage and analysis. The time between the onset of symptoms suggestive of malaria to presentation to the health centre was the primary outcome. The main exposure of interest was the response to the question ‘do you think that HIV testing is done for all people who give blood samples for malaria testing at the heath facility?’ During analysis, the outcome variable was categorized into ‘early’ (≤2 days) and ‘late’ (≤3 days) as used in previous studies of this topic [7,17-19] and also as categorization using this cut-off approximated to the median value. Initial analysis was by chi-squared testing and subsequent analysis by logistic regression adjusting for potential confounding factors and clustering by health centre. The impact of urban and rural locations on the primary exposure was explored as the prevalence of HIV infection varied between these settings. All demographic exposures of interest were considered as potential confounding factors if they modified the size of association between the primary outcome and the exposure by 10% or more. The population attributable fraction of the impact of the associations observed at the population level was calculated after converting odds ratios (ORs) to relative risks (RRs) [20,21].

The aim was to recruit 850 individuals. All children under the age of 16 years who presented at the health centre with their guardian and were clinically suspected of potentially having malaria (defined as having a blood film test requested) were eligible for inclusion in the study. The total sample size allocated to each health centre was proportional to the total patients tested for malaria in the previous 3 months (June-August, 2012) and individuals were recruited consecutively until the estimated sample size was achieved. Assuming that 20% of guardians had concerns about HIV testing in population and 50% of children have had symptoms for 5 days or less, it was estimated that the study would have over 90% power (5% risk of type I error) to detect an absolute increase of 20% in the guardians who had concerns about HIV testing in the children who experienced malaria symptoms for five days or more compared to those whose guardians had no such concerns.

### Role of the funding source

The sponsors of the study had no role in study design, data collection, data analysis, data interpretation, or writing of the report. The corresponding author had full access to the data in the study and had full responsibility for the decision to submit.

## Results

### Study demographics

A total of 836 guardians were invited to participate in the study and of these, 830 (99%) provided data for analysis. The demographics of the study participants are reported in Table 1. Some 400 children (48%) were reported as having symptoms of suspected malaria lasting more than 2 days, and 114 (14%) had sought health advice prior to the current presentation (Table 2). The overall prevalence of malaria was 20% for participants in the study, and the prevalence for each health centre was Modjo (12%), Meki (34%), Batu (18), Bulbula (15%), and Shashemenne (18%).

**Table 1:** Demographic characteristics of guardians

	**Total**	**Concern about covert HIV testing**		
	**(%) N = 830**	**Yes (%) N = 423**	**No/do not know (%) N = 407**	p value*
Location				
Urban	469 (57)	226 (53)	243 (60)	0.07
Rural	361 (43)	197 (47)	164 (40)	
Sex				
Male	263 (32)	133 (31)	130 (32)	0.88
Female	567 (68)	290 (69)	277 (68)	
Age (years)				
16-24	157 (19)	81 (19)	76 (19)	0.54
25-34	382 (46)	203 (48)	179 (44)	
35-44	231 (28)	112 (26)	119 (29)	
44+	60 (7)	27 (6)	33 (8)	
Relationship to sick child				
Mother	503 (61)	255 (60)	248 (61)	
Father	213 (26)	104 (25)	109 (27)	0.60
Sibling	78 (9)	45 (11)	33 (8)	
Other	36 (4)	19 (4)	17 (4)	
Highest level of education				
Illiterate	284 (34)	146 (35)	138 (34)	0.09
Literate but no formal education	69 (8)	30 (7)	39 (10)	
Primary school	302 (36)	168 (40)	134 (33)	
Secondary school or higher	175 (21)	79 (19)	96 (24)	
Main material of household’s roof				
Corrugated iron	614 (74)	306 (72)	308 (76)	0.27
Thatched	216 (26)	117 (28)	99 (24)	
Electricity in household				
Yes	507 (61)	250 (59)	257 (63)	0.23
No	323 (39)	173 (41)	150 (37)	
Perceived walking time to nearest health centre (min)				
<30	446 (54)	228 (54)	218 (54)	0.37
30-60	216 (26)	111 (26)	105 (26)	
60-120	91 (11)	41 (10)	50 (12)	
120-180	48 (6)	30 (7)	18 (4)	
>180	29 (3)	13 (3)	16 (4)	

*Chi-squared test.

**Table 2:** Details of child and history of suspected malarial symptoms

	**Total**	**Location**		
		**Urban**	**Rural**	p value*
Median age of child (IQR)	6 (2–9)	5 (2–9)	6 (3–9)	-
Sex of child (%)				-
Male	423 (51)	248 (53)	175 (48)	
Female	407 (49)	221 (47)	186 (52)	
Number of days of illness prior to health centre attendance				
<1	132 (16)	103 (22)	29 (8)	<0.001
1	66 (8)	41 (9)	25 (7)	
2	232 (28)	122 (26)	110 (34)	
3	236 (28)	124 (26)	112 (31)	
4 or more	164 (20)	79 (17)	85 (24)	
Main reason for delayed attendance N = 626, (%)				
Usually wait and see	187 (30)	107 (33)	80 (26)	<0.001
Not serious illness	174 (28)	103 (32)	71 (23)	
Lack of money	139 (22)	56 (17)	83 (27)	
No nearby health facility	47 (8)	8 (2)	39 (13)	
Fear of HIV testing	37 (6)	20 (6)	17 (6)	
Other	42 (7)	27 (8)	15 (5)	
Sought health advice prior to visiting current health centre (%)				
Yes	114 (14)	52 (11)	62 (17)	0.01
No	716 (86)	417 (89)	299 (83)	
Place of advice sought, N = 114, (%)				
Health post/centre	59 (52)	9 (17)	50 (81)	<0.001
Private clinic	44 (39)	35 (67)	9 (15)	
Pharmacy	10 (9)	8 (15)	2 (3)	
Other	1 (1)	0 (0)	1 (2)	

*Chi-squared test.

IQR = interquartile range.

### Concerns about HIV testing

Four-hundred and twenty-three (51%) individuals considered that HIV testing was applied to blood given for malaria testing at the health centre (Table 3); 353 (42%) individuals knew of people in the community who delayed seeking healthcare for malaria testing due to concerns about HIV testing; 242 (29%) of individuals considered that fear of HIV testing delayed presentation with suspected malaria, with a higher proportion of these, 160 individuals (34%), living in an urban environment compared to 82 participants (23%) who lived in a rural setting (p < 0.001).

**Table 3:** Health beliefs of guardians

	**Total**	**Home location**		
	**(%)**	**Urban**	**Rural**	p value*
Has opinion that HIV testing is done for all people who give blood for malaria testing at heath facility				
Yes	423 (51)	226 (48)	197 (55)	0.17
No	351 (42)	208 (44)	143 (40)	
Do not know	56 (7)	35 (7)	21 (6)	
Confidence that HIV testing is done on blood given for malaria testing				
Not at all/little sure	370 (45)	219 (47)	151 (42)	0.18
Somewhat sure	129 (16)	62 (13)	67 (19)	
Very/completely sure	295 (36)	168 (36)	127 (35)	
Do not know	36 (4)	20 (4)	16 (4)	
Aware of person in community who did not attend health facility for malaria testing due to fear of HIV test				
Yes	353 (43)	194 (41)	159 (44)	0.23
No	371 (45)	207 (44)	164 (45)	
Do not know	106 (13)	68 (14)	38 (11)	
Do you think that the fear of HIV testing is the reason for the delay in people seeking an early diagnosis of malaria at health facility?				
Yes	242 (29)	160 (34)	82 (23)	<0.001
No	386 (47)	192 (41)	194 (54)	
Do not know	202 (24)	117 (25)	85 (24)	

*Chi-squared test.

Children whose guardians suspected that blood provided for malaria diagnosis was covertly tested for HIV had a median delay of three days from the start of their symptoms to clinical evaluation compared to a median of two days for those children whose guardians did not hold this belief (p < 0.001). After adjustment for clustering by health centre, those children whose guardians were concerned about covert HIV testing had a higher odds of a prolonged delay before being seen at a health centre (OR 1.73, 95% CI: 1.10 to 2.70 for a delay of more than twp days). The age and sex of the child, residence, age of the guardian, main material of the household’s roof, presence of electricity, time to travel to the health centre and health centre location were not confounding factors for this association between HIV beliefs and a delay in presentation with suspected malaria. This resulted in a population attributable fraction for the association of concerns about covert HIV testing on delayed presentation with malarial symptoms in children of 14% (95% CI: 3 to 23%).

## Discussion

This is the first study to specifically assess the impact of concerns about HIV testing on the time to attendance at a health centre among children with suspected malaria. The data demonstrate that in this population, over half of guardians of children with suspected malaria considered that HIV testing was implemented on all blood donated for malaria testing and that 43% of respondents knew of others in their community who had delayed seeking medical care because of these concerns. These beliefs appear to impact not only on the individual, but their children as guardians who were concerned about HIV testing were more likely to bring their children for medical assessment later than those who had no such concerns.

### Methodological considerations and limitations

The study has a number of strengths including the prospective assessment of an *a priori* hypothesis from a population of the guardians of children with suspected malaria, who had no knowledge of the hypothesis being tested. The response rate was high at 99%, giving confidence in the generalizability of the findings to this population and the minimization of bias, and data were collected on co-variates permitting adjustment for other demographic factors that may have been important.

The main limitation of these data is that while they may be generalizable to the community studied during the period of high malarial prevalence, these attitudes to HIV testing and accessing health care in general are not necessarily applicable to other communities and cultures or at other times of the year. Hence, further studies are required to determine how prevalent the belief that covert HIV testing may accompany testing for malaria actually is in the many countries with a high prevalence of HIV. A delay of over two days before obtaining medical assessment after symptoms of suspected malaria started was seen in 49% of the study population, and similar delays have been observed from other observational studies from Nigeria [22], Senegal [23], Ethiopia [7,24] and India [19,25]. The estimates that these data provide of the impact of fear of covert HIV testing necessarily do not include those who died before presentation with malaria, and hence may potentially underestimate the overall impact of these beliefs on time to presentation. The data are cross-sectional, and hence it is not possible to demonstrate a causal effect. The authors were unable to exclude the possibility that acquiescence bias (an increased tendency to respond affirmatively to closed questions [26]) may have influenced the data, and await future studies that explore these beliefs and their impact on healthcare seeking behaviour in similar populations. Population-based cohort studies are necessary to study these hypotheses prospectively with consideration of baseline attitudes to HIV testing and observation of subsequent medical consultation rates. These should consider a broader range of diseases than just malaria as it is possible that these observations may reflect a wider mistrust of clinical care services that is delaying presentation of patients with other treatable conditions.

The prevalence of refusal for HIV testing among women attending for antenatal care in the Gambella Region of Ethiopia in 2008 is 25%, suggesting that in this population there was a sizeable minority who did not want to be aware of their HIV status [13]. The data suggest that these beliefs are adversely impacting on the presentation of children with suspected malaria for medical attention, due to the misplaced belief that they will also be tested for HIV. Surprisingly, little data exist on how attitudes to HIV may impact on presentation with potential malaria. Data from rural Côte d’Ivoire collected from 2010 of 100 individuals who were offered a rapid diagnostic testing for malaria reported that 67% considered that the blood would be used for HIV testing [27]. The only further data available are from two separate qualitative studies exploring the feasibility of introducing rapid diagnostic tests for malaria in Uganda in a variety of settings. In one qualitative study, community members expressed concern that blood samples collected for malaria could be used for HIV testing rather than malaria [15], which was similar to the belief held by 51% of the study population. In the second qualitative study, there were concerns that ‘in most communities people think that taking of blood means testing for HIV and some do not want their HIV status disclosed’ [16].

### Implications for policy

The observation that concerns about HIV testing may delay malaria diagnosis in children is important for public health policy makers as firstly malaria is a common disease that infects approximately 200 million individuals annually [1], and that secondly it is well recognized that prompt diagnosis and treatment are important to reduce morbidity and mortality [28-30]. As knowledge about the disease is amenable to public education programmes [31,32], delayed presentation with suspected malaria by the erroneous concern that covert HIV testing may also be performed may be considered a risk environmental factor that is amenable to educational programmes. If this association was causal, the OR of 1.73 approximates to a rate ratio of 1.32 [20,21], giving a population attributable risk fraction for the fear of covert HIV testing resulting in delayed presentation of 14% children with malarial symptoms.

## Conclusions

These data are consistent with the hypothesis that guardians’ concerns about covert HIV testing without informed consent is an independent risk factor for delayed presentation to health centres for children with suspected malaria. This is important, as it is a belief that is open to modification by appropriate intervention programmes, hence improving the early delivery of appropriate anti-malarial treatments to patients and hence improving clinical outcomes.

## Competing interests

The authors have declared that there are no competing interests.

## Authors’ contributions

YH was involved in proposal writing, designed the study and participated in coordination, supervision and the overall implementation of the project, analysed the data, drafted and finalized the manuscript. WD, AWF and GD conceived the study and participated in all stages of the study and revision of the manuscript. AWF obtained funding for the study and checked the statistical analyses. All authors read and approved the final version of the manuscript.

## Supplementary Material

Additional file 1 Study Questionnaire. Click here for file

Additional file 2 Strobe Statement. Click here for file

## References

[B1] WHO World Malaria Report 2013 2013 Geneva: World Health Organization

[B2] President’s Malaria Initiatives (PMIs) Malaria Operational Plan 2013 Addis Ababa, Ethiopia http://www.pmi.gov/docs/default-source/default-document-library/malaria-operational-plans/fy13/ethiopia_mop_fy13.pdf?sfvrsn=6 (accessed 28/12/2014)

[B3] Deressa W Hailemariam D Ahmed A Economic costs of epidemic malaria to households in rural Ethiopia Trop Med Int Health 2007 12 1148 1156 10.1111/j.1365-3156.2007.01901.x 17956496

[B4] White N Pukrittayakamee S Hien T Faiz M Mokuolu O Dondorp A Malaria Lancet 2014 383 723 735 10.1016/S0140-6736(13)60024-0 23953767

[B5] Bisoffi Z Sirima BS Angheben A Lodesani C Gobbi F Tinto H Van den Ende J Rapid malaria diagnostic tests vs clinical management of malaria in rural Burkina Faso: safety and effect on clinical decisions. A randomised trial Trop Med Int Health 2009 14 491 498 10.1111/j.1365-3156.2009.02246.x 19222821

[B6] Ahorlu C Koram K Ahorlu C de Savigny D Weiss M Socio-cultural deteminants of treatment delay for childhood malaria in southern Ghana Trop Med Int Health 2006 11 1022 1031 10.1111/j.1365-3156.2006.01660.x 16827703

[B7] Deressa W Chibsa S Olana D Treatment seeking of malaria patients in East Shewa Zone of Oromia, Ethiopia Ethiopian J Health Dev 2003 17 9 15

[B8] Chuma J Anbuya T Memusi D Juma E Akwale W Ntwiga J Nyandigisi A Tetteh G Shretta R Amin A Reviewing the lierature on access to proompt and effective malaris treatment in Kenya: implications for meeting Abuja targets Malar J 2009 8 243 10.1186/1475-2875-8-243 19863788 PMC2773788

[B9] UNAIDS Country progress report on HIV/AIDS response 2012 Federal Democratic Republic of Ethiopia: UNAIDS http://www.unaids.org/en/dataanalysis/knowyourresponse/countryprogressreports/2012countries/GAP%20Report%202012.pdf (accessed 28/02/2014)

[B10] Rankin W Brennan S Schell E Laviwa J Rankin S The stigma of being HIV-positive in Africa PLoS Med 2005 2 e247 10.1371/journal.pmed.0020247 16008508 PMC1176240

[B11] Greef M Uys L Wantland D Makoae L Chirwa M Diamini P Kohl T Mullen J Naidoo J Cuca Y Holzemer W Perceived HIV stigma and life satisfaction among persons living with HIV infection in five African countries: a longitudinal study Int J Nurs Stud 2010 47 475 486 10.1016/j.ijnurstu.2009.09.008 19854440 PMC2832093

[B12] Abaynew Y Deribew A Deribe K Factors associated with late presentation to HIV/AIDS care in South Wollo Zone Ethiopia: a case–control study AIDS Res Ther 2011 8 8 10.1186/1742-6405-8-8 21356115 PMC3058009

[B13] Fanta W Worku A Determinants for refusal of HIV testing among women attending for antenatal care in Gambella Region, Ethiopia Reprod Health 2012 9 8 10.1186/1742-4755-9-8 22834566 PMC3453494

[B14] Okello G Ndegwa S Halliday K Hanson K Brooker S Jones C Local perceptions of intermittent screening and treatment for malaria in school children on the south coast of Kenya Malar J 2012 11 185 10.1186/1475-2875-11-185 22681850 PMC3422207

[B15] Mukanga D Tibenderana J Kiguli J Pariyo G Waiswa P Bajunirwe F Mutamba B Counihan H Ojiambo G Källander K Community acceptibility of use of rapid diagnostic tests for malaria by community health workers in Uganda Malar J 2010 9 203 10.1186/1475-2875-9-203 20626863 PMC2914066

[B16] Mbonye AK Ndyomugyenyi R Turinde A Magnussen P Clarke S Chandler C The feasibility of introducing rapid diagnostic tests for malaria in drug shops in Uganda Malar J 2010 9 367 10.1186/1475-2875-9-367 21176131 PMC3020157

[B17] Chuma J Okungu V Molyneux C Barriers to prompt and effective malaria treatment among the poorest population in Kenya Malar J 2010 9 144 10.1186/1475-2875-9-144 20507555 PMC2892503

[B18] Ogunlesi T Okeniji J Oyedeji G Oyedeji O The influence of maternal socioeconomic status on the management of malaria in their children: implications for the ‘Roll Back Malaria’ initiative Niger J Paediatr 2005 32 40 46

[B19] Das A Ravindran T Factors affecting treatment-seeking for febrile illness in a malaria endemic block in Boudh district, Orissa, India: policy implications for malaria control Malar J 2010 9 377 10.1186/1475-2875-9-377 21192825 PMC3224374

[B20] Grant R Converting an odds ratio to a range of plausible relative risks for better communication of research findings BMJ 2014 348 f7450 10.1136/bmj.f7450 24464277

[B21] Zocchetti C Consonni D Bertazzi P Relationship between prevalence rate ratios and odds ratios in cross-sectional studies Int J Epidemiol 1997 26 220 223 10.1093/ije/26.1.220 9126523

[B22] Agu A Nwojiji J Childhood malaria: mothers” perception and treatment-seeking behaviour in a community in Ebonyi State, South East Nigeria J Comm Med Prim Health Care 2005 17 45 50

[B23] Smith L Bruce J Gueye L Helou A Diallo R Gueye B Jones C Webster J From fever to anti-malarial: the treatment-seeking process from rural Senegal Malar J 2010 9 333 10.1186/1475-2875-9-333 21092176 PMC3000420

[B24] Paulander J Olsson H Lemma H Getachew A San Sebastian M Knowledge, attitudes and practice about malaria in rural Tigray, Ethiopia Global Health Action 2009 2 doi:10.3402/gha.v3402i3400.1839 10.3402/gha.v2i0.1839 PMC2779931 20027277

[B25] Matta S Khotar A Sachdev T Assessment of knowledge about malaria among patients reported with fever: a hospital-based study J Vect Borne Dis 2004 41 27 31 15332483

[B26] Hinz A Michalski D Schwarz R Herzberg P The acquiescence effect in responding to a questionnaire Psychosocial Med 2007 **4**, PMC2736523 PMC2736523 19742288

[B27] Comoe C Outattara A Raso G Tanner M Utzinger J Koudou B Willingness to use a rapid diagnostic test for malaria in a rural area of central Côte d”Ivoire BMC Pub Health 2012 1012 1089 23249239 10.1186/1471-2458-12-1089 PMC3543836

[B28] McGready R Boel M Rijken M Ashley E Cho T Moo O Pimanpanarak M Hkirijareon L Carrara V Lwin K Phyo A Turner C Chu C van Vugt M Price R Luxemburger C Kuile F Proux S Singhasivanon P White N Nosten F Effect of early detection and treatment on malaria related maternal mortality on the North-Western border of Thailand, 1986–2010 PLoS One 2012 7 e40244 10.1371/journal.pone.0040244 22815732 PMC3399834

[B29] Durrheim D Frieremans S Kruger P Mabuza A de Bruyn J Confidental enquiry into malaria deaths Bull World Health Organ 1999 77 263 266 10212518 PMC2557624

[B30] Ejov M Tun T Aung S Lwin S Sein K Hospital-based study of severe malaria and associated deaths in Myanmar Bull World Health Organ 1999 77 310 314 10327709 PMC2557645

[B31] Rhee M Sissoko M Perry S Mcfarland W Parsonnet J Doumbo O Use of insecticide-treated nets (ITNs) following a malaria edadication intervention in Piron, Mali: a control trial with systematic allocation of households Malar J 2005 4 35 10.1186/1475-2875-4-35 16042793 PMC1208942

[B32] Ayi I Nonaka D Adjovu J Hanafusa S Jimba M Bosompem K Mizoue T Takeuchi T Boakye D Kobayashi J School-based participatory health education for malaria control in Ghana: engaging children as health messengers Malar J 2010 9 98 10.1186/1475-2875-9-98 20398416 PMC2865503

