# Supplementary material for: Concerns about covert HIV testing are associated with delayed presentation of suspected malaria in Ethiopian children: a cross-sectional study
Source: Malar J. 2014 Aug 6;13:301. doi: 10.1186/1475-2875-13-301 (PMC4126071; doi:10.1186/1475-2875-13-301)
Supplement: Additional file 1 — Study Questionnaire. [file 1475-2875-13-301-S1.docx]

**Annex-II QUESTIONNAIRE DESIGNED TO ASSESS FEAR OF HIV TESTING ON EARLY PRESENTATION AND TREATMENT OF MALARIA AMONG CHILDREN IN EAST SHEWA ZONE OF OROMIA REGION, ETHIOPIA**

1. **Quantitative part**

Questionnaire No: _______________________________________

Zone:______________________________ Woreda:______________________________

Name of the health center-----------------------------------------------------

1. **Individual information sheet**

Hello, my name is............................................ . I work for an institution named Addis Ababa University School of Public Health. I am one of the data collectors on the study with the above topic. I would like you to cooperate in answering the questions that follow. The information you will provide contributes to measures that are taken to control malaria.

Any information you provide will be confidential. You have the right to not to participate in the study.

Name of Interviewer: __________________________________ Date: _ _/_ _/_ _

Start time: _ _/_ _ End time: _ _/_ _

1. **Interviewer agreement**

‘I certify that I have filled this questionnaire in accordance with the training I was given and instructions stated in it. I have confirmed that the information in it is correct.’

Signed___________________________ date ________________________________

Date-----------------------------------/month---------------------------------/year----------------------------

Name of data collector--------------------------- signature----------------------------------------------

Interview date _ _/_ _/_ _

Name of supervisor---------------------------------signature----------------------------------------------

Date: _______________________

Are you willing to participate in the study?

Yes/No

Thank you

Concern about HIV test of caretakers

| **No** | Questions and filters | Response categories | Skip |
| --- | --- | --- | --- |
| 101 | Do you think that the blood drawn for malaria diagnosis will also be used for HIV test in the health facility? | Yes 1  No 2 |  |

**Section I: Socio-demographic Characteristics of the Respondents**

| No | Questions and filters | Response categories | Skip |
| --- | --- | --- | --- |
| 1 | What is your place of residence (rural/ urban)? | Rural 1  Urban 2 |  |
| 2 | Sex of interviewee: | Female 1  Male 2 |  |
| 3 | What is your (respondent) age in full years? | /_____/ Year |  |
| 4 | What is your relationship to the sick child? | Mother 1  Father 2  Brother 3  Sister 4  Other (Specify)__________5 |  |
| 5 | What is the sex of head of the household? | Male 1  Female 2 |  |
| 6 | What is your current marital status? | Married 1  Never married (single) 2  Divorced 3  Widowed 4  Separated 5 |  |
| 7 | What is your religion? | Islam 1  Orthodox 2  Catholic 3  Protestant 4  Other(specify) _____________________5 |  |
| 8 | What is your ethnicity? | Oromo 1  Amhara 2  Guraghie 3 Kambata 4  Hadiya 5  Tigre 6  Other(specify) ___________________ 9 |  |
| 9 | What is the highest level of school or grade you have completed? | Unable to read or write 1  Can only read and write 2  Primary Cycle 1 (1-4) 3  Primary Cycle 2 (5-8) 4  Secondary (9-12) 5  TVET (10+1 or 10+2) 6  TVET (10+3 /Diploma) 7  University Degree or Above 8  Other (specify)___________________ 9 |  |
| 10 | What is your current main work/occupation?   - ***Choose only one response*** | Farmer 1  Housewife 2  Daily labourer 3  Government employee 4  NGO employee 5 Trader 6  Student 7  Other (specify)__________________ 8 |  |
| 11 | Does your household have:  Electricity?  A functional radio?  A functional television?  A functional telephone?  A functional refrigerator? | **Yes** **No**  Electricity 1 2  Functional RADIO 1 2  Functional TELEVISION 1 2  Functional TELEPHONE 1 2  Functional REFRIGERATOR 1 2 |  |
| 12 | What is the main material of the roof of house of the household? | Thatched………………….1  Corrugated iron 2  Other (Specify)__________________3 |  |
| 13 | What is the health care facility that is nearest to your home? | Health post 1  Health center 2  Public/private hospital 3  Private clinic 4  Don’t know 5  Other (specify)__________________ 6 |  |
| 14 | How far is this health center from your home? | Less than 30 minutes’ walk 1  30 minutes – under 1 hour walk 2  1 hour to under 2 hours walk 3  2 hours to under 3 hours walk 4  3 or more hours 5  Don’t know 98 |  |
| 15 | How many minutes does it take to walk to the health post where people in your community generally go to get malaria treatment? | Less than 30 minutes’ walk 1  30 minutes – under 1 hour walk 2  1 hour to under 2 hours walk 3  2 hours to under 3 hours walk 4  3 or more hours 5  Don’t know 98 |  |

**Section II:**  **Knowledge about malaria prevention and treatment**

| No | Questions and filters | Response categories | Skip |
| --- | --- | --- | --- |
| **16** | Do you consider malaria a major health problem in your community? | Yes 1  No 2  Don’t know 3 |  |
| **17** | In your opinion, what causes malaria?   - ***Multiple responses possible and circle all responses that apply*** - ***Probe for possible answers (Anything else?)*** | Mosquito bite 1  Protozoan parasite 2  Eating immature sugarcane 3  Eating maize stalk 4  Hunger (empty stomach) 5  Exposure to cold or changing weather 6  Drinking dirty water 7  Witchcraft 8  Exposure to dirty swampy areas 9  Through body contact with malaria patient 10  Don’t know 11  Other (specify) ________________ 12 |  |
| **18** | What are the main signs and symptoms of malaria?   - ***Multiple responses possible and circle all responses that apply*** - ***Probe for possible answers (Anything else?)*** | **Yes No**  Fever 1 2  Feeling cold 1 2  Sweating 1 2  Headache 1 2  Nausea 1 2  Vomiting 1 2  Loss of appetite 1 2  Bitterness in the mouth 1 2  Body weakness/tiredness 1 2  Body ache/joint pain 1 2  Thirsty 1 2  Diarrhea 1 2  Don't know 1 2  Other (specify)___________________ |  |
| **19** | For which group of the population do you think malaria is more serious?   - ***Multiple responses possible and circle all responses that apply*** - ***Probe for possible answers (Anything else?)*** | Adults ……. 1  Children under 5 years of age…. 2  Children ……. 3  Pregnant women……. 4  Elderly ……. 5  Equally serious for all……. 6  Don't know or not sure…. 7 |  |
| **20** | How can someone protect themselves against malaria?   - ***Multiple responses possible and circle all responses that apply*** - ***Probe for possible answers (Anything else?)*** | Sleep under a mosquito net/ITNs……1  Avoid mosquito bites……2  Spray house with insecticide……3  Drain mosquito breeding sites around the house 4  Keep house surroundings clean…..5  Smoking (burn leaves/cow dung) nearby the house .6  Don’t drink dirty water…..7  Don’t eat bad food (immature sugarcane/leftover food)….8  Put mosquito screens on the windows….9  Eat garlic….10  Drink alcohol…11  Other (Specify)__________________12  Don’t know 13 |  |
| **21** | Is malaria a preventable disease? | Yes 1  No 2  Don’t know 3 |  |
| **22** | Is malaria a treatable/curable disease? | Yes 1  No 2  Don’t know or not sure 3 |  |
| **23** | What is the outcome of malaria if not treated earlier?   - ***Multiple responses possible and circle all responses that apply*** | Death 1  Self-cure 2  Disability 3  Don’t know 4  Others (specify)_____________5 |  |
| **24** | What is the current drug of choice for treatment of malaria?   - ***Multiple responses possible and circle all responses that apply*** | Chloroquine 1  CoArtem 2  Quinine 3  Fansidar 4  Don’t know 5  Other (specify)___________ 6 |  |
| **25** | Does your household currently have any mosquito nets that can be used while sleeping? | Yes 1  No 2 | ***If 1 to Q 26*** |
| **26** | How many mosquito nets/ITNs does your household currently have [both used and unused]? | /______/ |  |
| **27** | How frequently did you sleep under mosquito net/ITNs within the last 15 days? | All nights 1  Almost all nights 2  Sometimes 3  Only few nights 4  None of the nights 5 |  |
| **28** | Did you sleep under mosquito net/ITNs last night? | Yes 1  No 2 |  |
| **29** | How frequently did the sick CHILD sleep under mosquito net/ITNs within the last 15 days? | All nights 1  Almost all nights 2  Sometimes 3  Only few nights 4  None of the nights 5 |  |
| **30** | Did the sick CHILD sleep under mosquito net/ITNs last night? | Yes 1  No 2 |  |

**Section III: Knowledge about HIV/AIDS Prevention and Testing**

Treatment seeking behavior for a child with fever

| No | Questions and filters | Response categories | Skip |
| --- | --- | --- | --- |
| 31 | What is the sex of the child with fever? | Female 1  Male 2 |  |
| 32 | How old is the sick child in full years? | /_____/ Year |  |
| 33 | How many days ago did the fever start?   - ***If less than one day, record “00”*** | Days ago  Don’t know…..…………98 |  |
| 34 | What do you think with the cause of your child’s current illness? | Malaria 1  HIV/AIDS 2  Other (specify)___________3 |  |
| 35 | Did you seek advice or treatment for the child from any source before coming to this health center? | Yes 1  No 2 |  |
| 36 | Where did you seek advice or treatment for the child? Anywhere else?   - ***Record all sources mentioned*** | Health post 1  Health center 2  Private clinic 3  Pharmacy/drug shop 4  Govt/private hospital 5  Other (specify)_______6 |  |
| 37 | How many days after the fever began did you first seek advice or treatment for ***the child***?  If the same day, record “00” | Days _______ |  |
| 38 | At any time during the illness, did the child take any drugs for the fever? | Yes 1  No 2  Don’t know/not sure 3 |  |
| 39 | At any time during the illness, did ***the child*** take any ANTIMALARIAL DRUG? | Yes 1  No 2  Don’t know/not sure 3 | If 1 to Q40 |
| 40 | What ANTIMALARIAL DRUG did ***the child*** take?   - Probe (Any other drug?) - ***Record all responses*** | CoArtem 1  Chloroquine 2  Quinine 3  Fansidar 4  Don’t know 9  Other (specify) _______ 10 |  |
| 41 | What was the reason for coming to this health facility? | Condition becoming worse 1  Referral from other health  personnel 2  To get malaria testing 3  Others (specify)_________4 |  |
| 42 | How many days after the start of the illness did the child come to this health center?  If the same day, record “00” | Days _______ |  |
| 43 | If the duration for Q 42 is >1day, what was the main reason for the delay in seeking treatment? | Lack of money 1  Fear of HIV testing 2  Not serious illness 3  No nearby health facilities 4  Usually first wait  and see 5  Others (specify)________ 6 |  |
| 44 | Do you think that the health professionals will check the HIV status of people from the blood sample collected for malaria testing, without consent? | Yes 1  No 2  Don’t know 3 |  |
| 45 | If yes to Q44, is this a main reason for delaying early presentation and treatment of malaria at health facilities? | Yes 1  No 2 |  |

**Section IV: Concerns about HIV testing in delaying early presentation and treatment for malaria**

| No | Questions and filters | Response categories | Skip |
| --- | --- | --- | --- |
| 46 | Is there any test that a person can take to determine if he (or she) has malaria? | Yes 1  No 2  Don’t know/not sure 3 |  |
| 47 | Is there any test that a person can take to determine if he (or she) has HIV, the virus that causes AIDS? | Yes 1  No 2  Don’t know/not sure 3 |  |
| 48 | Is blood test for malaria and HIV similar**?** | Yes 1  No 2  Don’t know/not sure 3 |  |
| 49 | HIV testing is done for all people who gave their blood sample for malaria testing at health facility? | Yes 1  No 2  Don’t know/not sure 3 |  |
| 50 | How sure are you that you would get an HIV test if you give your blood sample for malaria testing at health facility? | Not at all sure 1  Only a little sure 2  Somewhat sure 3  Very sure 4  Completely sure 5  Don’t know/not sure 6 |  |
| 51 | How many people in your community believe that people who give blood sample for malaria test will also be tested for HIV? | None 1  Only few 2  Many 3  Almost all 4  All 5  Don’t know/not sure 6 |  |
| 52 | Have you heard of any person in your community who did not go to health facility for malaria testing due to fear of HIV test? | Yes 1  No 2  Don’t know/not sure 3 |  |
| 53 | Do you think that the health professionals will check your child’s HIV status from the blood sample collected for malaria testing, without your consent? | Yes 1  No 2  Do not know 3 | If 1 to Q54 |
| 54 | Is this a main reason for delaying early presentation and treatment of malaria at health facilities? | Yes 1  No 2 |  |
| 55 | Are you comfortable with HIV test? | Yes 1 No 2 | If 2 to Q 56 |
| 56 | Why don’t you interested?   - ***More than one answer possible*** - ***Encircle all that apply*** | Because I do not expect HIV in my child 1  Because I fear that the information may not be secret 2  Because the test is painful for my child 3 Other(specify)______________________4 |  |

**Section V: Laboratory results of the child with fever**

| No | Questions and filters | Response categories | Skip |
| --- | --- | --- | --- |
| 57 | Blood film was taken for the child with fever? | Yes 1  No 2 | If 1 to Q58 |
| 58 | Blood testing of the child was done using: | Microscopy 1  RDT 2 |  |
| 59 | What was the lab test result of the child? | Positive 1  Negative 2 | If 1 to Q60 |
| 60 | What was the *Plasmodium s*pecies responsible for the illness? | P. falciparum 1  P. vivax 2  Mixed of both 3  Other (specify)_______4 |  |
| 61 | Did the child being prescribed any medication? | Yes 1  No 2 |  |
| 62 | Did ***the child*** being prescribed any ANTIMALARIAL DRUG? | Yes 1  No 2  Don’t know/not sure 3 | If 1to Q63 |
| 63 | What ANTIMALARIAL DRUG did ***the child*** take?   - Probe (Any other drug?) - ***Record all responses*** | CoArtem 1  Chloroquine 2  Quinine 3  Fansidar 4  Don’t know 9  Other (specify) _______ 10 |  |

***Thank you for your response!!!***
